# GP perspectives on a computer-assisted strategy to support PPI deprescribing: a qualitative study

**DOI:** 10.1038/s41598-026-41970-w

**Published:** 2026-03-11

**Authors:** Nele Kornder, Bettina Bücker, Alexandra Schmidt, Wiebke Reichert, Susanne Löscher, Michaela Maas, Anne Barzel, Annette Becker, Stefan Wilm, Annika Viniol, Julia Heisig

**Affiliations:** 1 Department of Primary Care, Marburg University, Karl-Von Frisch-Str. 4, 35043 Marburg, Germany; 2 Institute of General Practice (Ifam), Centre for Health and Society (Chs), Medical Faculty, Heinrich Heine University Düsseldorf, Düsseldorf, Germany; 3 Chair of General Practice II and Patient-Centeredness in Primary Care, Institute of General Practice and Primary Care (IGPPC), Faculty of Health, Witten/Herdecke University, Witten, Germany; 4 Department of General Practice and Primary Care, Ulm University Hospital, Ulm, Germany

**Keywords:** Proton pump inhibitor, Deprescribing, Decision support system, Family medicine, Health care, Medical research

## Abstract

Proton pump inhibitors (PPIs) are widely prescribed in primary care but often continued longer than clinically necessary, exposing patients to avoidable risks. Digital decision-support tools have been proposed to assist clinicians in identifying and managing potentially inappropriate medications. The arriba-PPI tool was developed to facilitate conversations on PPI deprescribing between general practitioners (GPs) and patients, supporting evidence-based and shared decision-making. To explore GPs’ experiences with the arriba-PPI tool in clinical practice and understand factors influencing its use and impact on PPI prescribing and discontinuation. A qualitative exploratory study embedded within a multicentre cluster-randomised controlled trial conducted in German general practices. Semi-structured interviews were conducted with 26 GPs from the intervention arm who had used the tool. Interviews were analysed following Braun and Clarke’s six-step thematic analysis methodology, applying a combined deductive–inductive approach. Six main themes emerged: perceived usefulness and acceptance of the tool; tool functionality and areas for improvement; patient perspectives and characteristics; doctor–patient interaction and consultation dynamics; PPI prescribing and discontinuation practices; and implementation context and long-term use. GPs valued the tool’s structured format and visual aids for enhancing communication, particularly with patients reluctant to stop PPIs. Some described an educational benefit, reporting greater awareness and reflection on their own prescribing behaviour. Barriers to sustained use included technical issues, workflow integration challenges, and the absence of non-pharmacological alternatives. Trust and established doctor–patient relationships were seen as critical for successful deprescribing, often surpassing the tool’s direct influence. While the arriba-PPI tool supports deprescribing conversations, its effectiveness depends on seamless integration, technical optimisation, and complementary non-drug strategies. Future digital interventions should follow established frameworks for complex intervention development, adopt a more holistic approach, and combine technological support with broader patient-centred care to achieve sustained deprescribing success in primary care.

**Supplementary Information:**

The online version contains supplementary material available at 10.1038/s41598-026-41970-w.

## Introduction

### Clinical use and challenges of PPI prescribing

Proton pump inhibitors (PPIs) are commonly prescribed in general practice as a first-line treatment for gastritis, the prevention of Non-Steroidal Anti-Inflammatory Drugs (NSAIDs)-associated ulcers, gastroesophageal reflux disease (GERD), and other acid-related conditions such as erosive esophagitis^1^. PPIs are also used in combination with antibiotics to eradicate *Helicobacter pylori*. They are generally well tolerated, safe, and offer superior and longer-lasting acid suppression compared to other agents such as histamine-2 receptor antagonists^2^.

Despite their widespread use and favourable short-term safety profile, concerns have been raised regarding the long-term use of PPIs. Prolonged intake has been linked to impaired mineral absorption, potentially increasing the risk of fractures of the hip, wrist, and spine^3^. Additional risks include pneumonia, enteric infections such as *Clostridium difficile*, adverse drug interactions (e.g., with clopidogrel), and possible associations with cardiovascular disease and heart failure^4–7^.

In light of these potential harms, international guidelines recommend discontinuing PPI treatment after resolution of uncomplicated GERD, gastritis, gastroduodenal ulcers, or completion of *H. pylori* eradication therapy^8–10^.

Accordingly, PPI use should be regularly reviewed with the goal of prescribing the lowest effective dose, and deprescribing should be considered where clinically appropriate^11^.

However, deprescribing PPIs in clinical practice remains challenging. Patients often fear symptom recurrence or are reluctant to modify contributing lifestyle factors. At the same time, general practitioners (GPs) may continue PPI prescriptions initiated in hospital settings or may lack awareness of the potential harms associated with long-term use^12–14^.

### The arriba-PPI tool

The arriba software is a digital decision-support tool designed to promote shared decision-making between GPs and patients on various clinical topics^15^. It has been widely used in German general practice since 2010, for example, the module for cardiovascular prevention (arriba-KVP). A newer module, arriba-PPI, was developed to address the issue of long-term and potentially inappropriate PPI prescribing, using an iterative, interdisciplinary approach involving GPs, researchers, and software developers to ensure clinical relevance and usability in primary care.

Arriba-PPI provides evidence-based information to GPs regarding PPI indications and guides the consultation process. After entering patient-specific data, the tool visually displays a “traffic light” recommendation: green indicating discontinuation is reasonable, amber suggesting a potential discontinuation and red advising against discontinuation. A visual scale outlines pros and cons to support discussion, and a printed handout offers patients information on tapering strategies and symptom control options.

Following its development, the module was evaluated in a multicenter, cluster-randomized controlled trial (RCT)^16^. In the intervention group, GPs received training via a 10-min instructional video on shared decision-making, discontinuation strategies, and the use of the tool (https://arriba-hausarzt.de/module/ppi-protonenpumpen-hemmer-absetzen). GPs in control practices were instructed to continue usual care for their study patients and did not receive access to the arriba-PPI tool, training, or any additional study-related support.

In 49.2% of consultations using arriba-PPI, a shared decision was made to reduce or discontinue PPI therapy. After six months, the intervention group showed a significant 22.3% reduction in the defined daily dose (DDD) of prescribed PPIs compared to the control group (95% CI 18.55 to 25.98; *p* < 0.0001).

### Objectives of the qualitative study

In order to improve comprehension in applying the intervention in everyday practice, a qualitative study was conducted. The aim was to explore the mechanisms behind the observed effects, identify unanticipated influences, and capture the subjective experiences of those involved.

This substudy, alongside the qualitative study by Schmidt et al.^17^, is part of the process evaluation of the complex arriba-PPI intervention^18^. It provides insight into the practical use of the tool, experiences during counseling about PPI use, and challenges encountered when attempting to reduce or discontinue PPI therapy.

The research questions are:

1. How did physicians perceive the arriba-PPI tool and its use in consultation situations?
2. What were the experiences with the tool?
3. How did physicians experience communicating with their patients about PPI?

## Methods

### Study design

We performed an exploratory study using semi-structured interviews to gain deeper insights into GPs experiences, motivations and opinions. The study reports according the COREQ guidelines^19^. We obtained informed consent from all participants. The protocol followed the tenets of the Declaration of Helsinki.

### Research team and reflexivity

A multi-disciplinary team from physicians, psychologists and health care researchers developed a semi-structured interview guideline. BB is a physician (MD) and AS has a MA in social science, they were both working as research associates at the time of the study with several years’ experience in qualitative research. The guideline was reflected multiple times in group discussions with all authors. JH has a PhD in biology and NK is a general practitioner (MD), they are both research associates with several years’ experience with qualitative studies.

Three research assistants with a bachelor’s degree (in biotechnology, medicine and information science) conducted the interviews. BB trained them in interview conduction techniques. In addition, interviewers received feedback from experienced qualitative researchers based on their audio recordings. Although the interviewees took part in the intervention group of the arriba-PPI RCT, they did not personally know the research assistants conducting the interviews. The interviewers indicated their role as a member of the research team who installed the arriba-PPI tool on their computers and who introduced them to the background of PPI deprescribing at least six months prior to the interviews.

WR who coded the transcripts has a bachelor’s degree in medicine and has been a research assistant at the primary care research institute for several years.

Research associates conducted three pretest interviews with GPs which resulted in modifications of the interview guide. The guide also included standardized instructions for opening the interview to ensure comparable conditions across interviewers.

### Sampling, recruitment and data collection

A total of 158 GPs from 144 general practices participated in the arriba-PPI multicenter cluster-RCT^16^ (Table 2). For the qualitative study, GPs were purposively sampled from those participating in the intervention group of the RCT to ensure an equal distribution across study sites. Research assistants at the University of Düsseldorf recruited GPs by phone. Beyond participation in the RCT and willingness to take part in an interview, no additional inclusion criteria were applied. GPs were contacted consecutively and interviewed until new interviews did not lead to new themes and sufficient data had been collected to answer the study question. Eight GPs could not be reached or declined to participate. Interviews were held over the phone and were completed in the time span from March to October 2020, with the majority of interviews conducted from March to May 2020.

As a narrative stimulus, the physicians were asked to recall their last patient conversation as part of the PPI project, in which they had applied the tool. Additionally, they were prompted to elaborate on said conversation spontaneously including emotions they were experiencing at that point in time. Main topics and interview questions are shown in Table 1. Prior to data collection, three pretest interviews were conducted, resulting in minor modifications of the interview guide. The guide also included standardized instructions for opening the interview to ensure comparable conditions across interviewers. Following each interview, we assessed whether new insights could be gained.

**Table 1:** Interview guide.

Interview guide
Main topic: Experience with arriba-PPI Question: Think back to the conversation you had with one of your patients in the PPI study, what was that like? Please tell me about the conversation.
Main topic: Shared-decision making Question: When you reflect on the situation, how did the patient contribute to the decision-making process?
Main topic: Reasons for using/not using arriba-PPI Question: What role does the tool play for you today in advising PPI patients? Do you still use the tool?
Main topic: General experience with arriba Question: Did you already use the arriba program before the study?
Main topic: Assessment of arriba-PPI Question: Was there anything in particular about the tool that helped or hindered the conversation with your patients? What wishes do you have for the further development of the tool?
Main topic: Handling of PPI intake in general Question: What would you like to see in the care of your PPI patients? What specific wish would you have?

Overview of main topics and corresponding interview questions.

### Analysis

Interviews were audio-recorded and additional field notes were taken. Transcription was completed verbatim and pseudonymously by an external service provider following the rules of Kuckartz^20^. We checked the transcripts randomly and compared them with the audio documents for quality assurance.

The material was analysed using reflexive thematic analysis following Braun and Clarke’s six-step methodology, applying a combined deductive–inductive approach. While the interview guide informed the initial orientation of coding (deductive), new codes and themes were developed directly from the data to allow for inductive insights. Team members (WR, JH, NK) familiarised themselves with the data through repeated reading and analytical note-taking. One researcher (WR) then conducted the systematic coding of the interviews. The remaining team members did not independently code all transcripts but contributed to the analysis through repeated familiarisation with the data and iterative, in-depth discussions of codes, themes, and interpretations. Codes and preliminary themes were developed collaboratively and were continuously and critically discussed within the research team, drawing on the expertise of members experienced in qualitative research. Initial codes were organised into meaningful themes, which were iteratively refined through regular, in-depth team discussions of codes, themes, and corresponding interview excerpts to ensure clarity, coherence, and distinctiveness of each theme. Coded segments were organised and visualised (e.g., in thematic maps and code hierarchies) to support the iterative refinement of themes and their underlying relationships. The qualitative data were analyzed using *MAXQDA 24* (VERBI Software, 2025). Language editing of the manuscript was supported by ChatGPT (OpenAI).

## Results

A sample of 26 GPs (out of 80 GPs) from the RCT intervention group was interviewed (Table 2). Data collection stopped after 26 interviews, as subsequent discussions yielded only repeated arguments and statements consistent with earlier ones, indicating that theoretical saturation had been reached. Interviews were conducted between 21 and 309 days after time point T1 (primary outcome assessment, 6 months after the GP training and introduction of the arriba-PPI tool in the respective practice) (mean: 143 days) .

**Table 2:** Number of GPs who participated in the multicenter cluster-RCT.

	All (n = 158)	Marburg	Witten	Düsseldorf
Control group	78	28	27	23
**Intervention group**	**80**	**37**	**24**	**19**
Interview participants	26	11	7	8

Only GPs from the intervention group (in bold) were recruited for the interviews.

Interviews were held between 21 and 309 days after time point T1 (= timepoint of primary outcome; 6 months after the GP’s advanced training) (mean: 143 days). The duration of the interviews ranged from 5 to 18 min (median: 9.5 min). Of the 26 GPs interviewed, 7 (27%) were female and 19 (73%) male (Table 3) (RCT: 33.5% female, 66.5% male; Germany: 50.7% female in 2023^21^). The median age was 58 years (36–65 years). About four fifths of the respondents were already aware of arriba before the study (overall study 75%), one third used other arriba modules regularly (overall study: one fourth). Individual demographic details and practice-specific durations between the introduction of arriba-PPI and the interviews are provided in Supplementary Table S1.

**Table 3:** Characteristics of interviewed GPs.

	n = 26*
Gender	
Women	7
Men	19
Study site	
Marburg	11
Witten	7
Düsseldorf	8
Age in years (median)°	36–65 (58)
Practical experience as GP in years (median)	3–37 (19.5)
Setting°°	
Rural	9
Urban	13
Type of practice	
Single	14
Group	10
Practice size^+^	
Small	3
Medium	9
Large	12
Familiar with arriba	19
Regular use of arriba°°	7

*24 questionnaires from 26 interviewed GPs.

°1 GP (°°2 GPs) did not answer this question.

^+^Practice size is determined by health insurance certificates per quarter; small: < 900 certificates, medium: 900–1500 certificates, large: > 1500 certificates.

### Content analysis resulted in six core categories

**Fig. 1:**
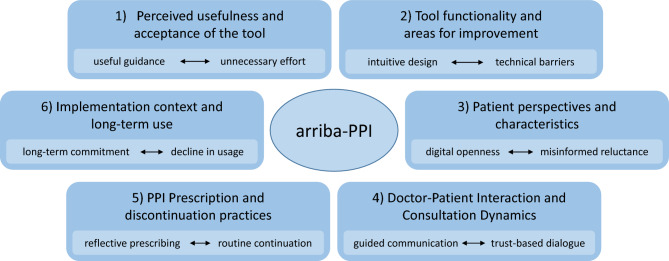
Six core categories.

Based on our qualitative analysis of the interview data, six overarching themes were identified that capture the participants’ experiences and perceptions related to the use of the decision-support tool in clinical practice (Fig. 1):

1. Perceived usefulness and acceptance of the tool
2. Tool functionality and areas for improvement
3. Patient perspectives and characteristics
4. Doctor-patient interaction and consultation dynamics
5. PPI prescription and discontinuation practices
6. Implementation context and long-term use

Each theme is further illustrated by related subthemes, which provide deeper insight into the varied ways in which the tool was perceived, integrated, and evaluated in daily primary care routines.

#### Perceived usefulness and acceptance of the tool

Participants reported mixed experiences regarding the tool’s usefulness.

While some found the tool helpful, others perceived it as only partially useful or even redundant, noting that it added little beyond routine consultation practices:

*“It doesn’t make things more difficult, but I didn’t find it particularly impressive either. There really isn’t that much to it. You can go through the individual pages with the patient, but in principle, the conversation itself is sufficient.”* (D2)

The tool’s visual elements—such as the traffic light system—were frequently highlighted as particularly helpful, especially for structuring consultations and supporting communication with patients who were otherwise difficult to persuade:

*“Then it was often very clear, yes, so either red or, or green."* (D7)

Several practitioners also emphasised its value for early-career clinicians, as it helped guide structured, evidence-informed decision-making:

*“Well, it is certainly the case that people with less experience, or doctors with less experience, can naturally better orientate themselves with something like that [the new tool] than someone who has been doing it forever."* (M3)

The tool was appreciated as a practical aid that could support clinical reasoning and streamline workflows, particularly in time-pressured settings. At the same time, some GPs noted drawbacks, reporting that the tool occasionally disrupted the flow of consultations and was used mainly because of study requirements

*“What the tool asks for is basically what I usually go through anyway. Since we are generally in a deprescribing mode, we really used the tool only for the purposes of the study, and I don’t see any use for it afterwards.”* (D2)

#### Tool functionality and areas for improvement

Despite perceived benefits, several functional limitations were reported. Some users described technical issues, lack of flexibility, and the additional time required to integrate the tool into their routines:

*“One disadvantage is partly the time burden having to open it separately to check it, to enter patient details, to doublecheck the patient’s current dosage, and so on.”* (D4)

Beyond these practical concerns, some GPs questioned the tool’s overall added value. They noted that its outputs often reiterated information they already knew, limiting its perceived usefulness:

*“...if you could calculate a score, ...then I might consider it. But as it is, it basically only gives me the answers I already know.”* (D6)

Suggested improvements included updating the tool’s instructions, particularly to provide clearer guidance on its appropriate use in complex clinical situations and in patients with specific indications where PPI therapy should not be modified. In addition, participants suggested offering more tailored outputs that better reflect individual patient characteristics, such as comorbidities, relevant indications, and contextual factors, as well as enhancing the tool’s visual design. Integration into existing practice management systems and linkage with other digital decision-support modules were also recommended, along with options for multilingual use and broader applicability to other medications.

The tool’s print function, which allows clinicians to generate a brief printed summary of the consultation for the patient at the end of the session, received mixed feedback. Some clinicians used this feature routinely for documentation or patient education, while others questioned its value due to limited patient interest. Preferences varied widely, depending on individual workflow and consultation content.

#### Patient perspectives and characteristics

Patient characteristics played a major role in how physicians perceived the relevance and usefulness of the tool in individual consultations. Patients with greater physician trust and less exposure to medical misinformation responded more positively, while “difficult” patients or those strongly attached to PPIs were more reluctant. Younger, digitally familiar patients benefited more from the tool.

*“The advantage is, as I said, that you can make it clear—especially to some computer-savvy and younger people—that the PPI is not necessarily required.”* (D4)

Moreover, participants noted different reasons for patients’ skepticism. Some, especially those who considered themselves particularly well-informed—often based on extensive internet research—remained critical regardless of the tool’s content:

*“Of course, as always, we also have the hypercritical patients, who subjectively consider themselves much better informed, and who just look at you with a friendly smile and say: Go ahead and talk.”* (W6)

This illustrates how prior attitudes and perceived knowledge can limit the tool’s persuasive potential. In contrast, one GP emphasized that the tool’s visual elements can be particularly helpful when dealing with patients who are difficult to persuade:

*"I use it with patients who are problematic—those who can’t be convinced through verbal explanation alone. In such cases, I think a traffic light system ultimately offers a different kind of perception. That’s when it makes sense, and then they say, ‘Okay, I can go along with that,’ or they’re willing to change things if necessary."* (M10)

#### Doctor-patient interaction and consultation dynamics

Physicians reported perceived changes in communication with patients when using the tool during consultations. Visual aids were seen as particularly helpful in making discussions more accessible and guiding the conversation. One physician noted:

*“For me, it was actually a good thing to guide the conversation more effectively—because you really had a clear thread to follow.”* (D5)

Some physicians used the tool to initiate or support tapering plans for PPIs, while others noted it as a basis for shared decision-making. However, in a few cases, the tool was perceived to reduce the patient’s active involvement. GPs emphasised often that patients could also be convinced through conversation alone, and that critical self-reflection by patients was often observed regardless of tool application. One physician highlighted the importance of long-standing doctor-patient relationships:

*“When you have patients, you’ve been seeing for years, where a relationship of trust has developed, and I explain to them that it’s basically not good to take PPIs for months or years, unless it’s absolutely necessary, they usually believe me.”* (M5)

This suggests that trust and continuity in care can be just as influential as digital interventions when discussing deprescribing. Several GPs emphasised that the established doctor–patient relationship was often far more decisive than the tool itself.

#### PPI prescription and discontinuation practices

The study revealed diverse practices around PPI prescription and discontinuation. While physicians reported that some patients were able to discontinue PPIs successfully, others continued despite lacking strict indications. The ease of prescribing PPIs and the lack of alternative non-pharmacological options were noted as structural and organisational challenges within routine primary care practice. Some GPs criticised that the tool focused primarily on reducing or removing medication without offering patients specific, actionable alternatives. One physician compared this to other digital tools:

*“That’s the difference compared to the cardiovascular risk calculator, which includes things like physical activity and smoking. You can show patients how the bars change—like, ‘If you stop smoking, this and that will happen.’ We don’t really have that in the PPI tool in the same way.”* (D2)

This physician further suggested integrating *“lifestyle indicators like diet, exercise—all that kind of stuff”* (D2) to enhance patient engagement and offer a more holistic approach to deprescribing.

At the same time, the study and tool appeared to raise awareness and professional reflection. GPs reported that tapering was often difficult and time-consuming, as many patients resisted due to symptom recurrence or long-standing habits. Education on the limited efficacy of PPIs in treating underlying conditions was considered essential for effective deprescribing. One physician emphasised that, overall, *“there is a sense that awareness of the issue has increased”* (D7). Another participant noted a personal shift in perspective, stating

*“Through the study alone, I’ve become significantly more critical of the whole issue.”* (W4)

However, not all GPs perceived a personal benefit from the tool itself. Some expressed confidence in their own clinical judgement and saw little added value:

*“Yes, for my own decision-making … I don’t need a tool for that.”* (M1)

This suggests that beyond patient communication, the intervention fostered deeper professional reflection and a reevaluation of prescribing practices, although its perceived necessity varied among GPs.

#### Implementation context and long-term use

After the study period, use of the tool declined and was generally infrequent. While a few GPs continued to use it occasionally, most discontinued its use due to factors such as time constraints, absence of suitable patient cases, technical issues (e.g., the tool not being reinstalled after a license renewal), and perceived redundancy after initial familiarisation. Several GPs noted that once the core message had been internalised, the tool offered little new information, reducing the need for continued active use:

*"I haven’t used it recently because I thought I had now covered the patients with whom I wanted to address the issue, so that’s been done. And for myself, I’ve more or less kept the indication in mind."* (D7)

Similarly, another GP emphasised that they had only used the tool within the study context and did not anticipate any further application afterwards:

*"So, what the tool asks for is also what I usually go by anyway, and since we are always in discontinuation mode anyway, we really only used the tool for the study, and I can’t see any use for it afterwards either."* (D2)

## Discussion

### Summary

Participants reported varying perceptions of the arriba-PPI tool’s usefulness. While some highlighted benefits for structuring consultations and supporting decisions, others reported limited added value, noting that the tool was sometimes used mainly because of study requirements and could even disrupt consultations. The tool influenced doctor-patient communication, particularly through visual elements, and played a role in PPI prescribing and discontinuation practices. Its effectiveness was shaped by patient characteristics, and several functional and technical limitations were noted. Long-term use depended on factors such as time constraints and integration into workflows.

### Comparison to existing literature

Interview findings showed ambivalent GP perceptions of the arriba-PPI tool, valuing its structure, especially for less experienced colleagues, while others saw little added value. Trust and communication emerged as central to consultations. Many GPs emphasised that patient motivation and willingness to discontinue PPIs were more strongly influenced by their relationship with the physician than by structured decision tools. This aligns with previous research that has identified the GP as a key figure in facilitating deprescribing conversations through trust-based dialogue rather than technological support^22–24^.

At the same time, GPs reported regular use of other shared decision-making modules. The preference seemed to hinge on the design and interactivity of the tool—factors that influenced both engagement and practical use. Tools such as arriba-KVP^25^, which offer more interactive graphics, were mentioned more favourably. This supports previous findings that even visually well-designed tools can be dismissed if they do not resonate with clinicians’ needs or workflow^26^.

A key barrier to acceptance was the lack of integrated non-pharmacological alternatives, as GPs were reluctant to recommend PPI discontinuation without options such as dietary advice, smoking cessation, or exercise, which limited the tool’s perceived relevance. This issue echoes broader findings in deprescribing research, which emphasise that discontinuation is more successful when embedded within a holistic, supportive care strategy^27,28^. In their qualitative study about professional roles in collaborative medication deprescribing, Gerlach et al. found that there is a “lack of positive reinforcements to deprescribing activities for GPs” to resort to^22^. Addressing this gap could enhance the tool’s perceived value and usability in practice.

Furthermore, the narrow scope of the tool—focusing solely on PPIs—may have contributed to its limited long-term use. This raises the question of whether the tool might be more effective as an educational resource or if it should be expanded into a more comprehensive platform targeting various potentially inappropriate medications**.** One promising development in this direction is the “MediQuit” tool, which seeks to support deprescribing across different drug classes^29^. However, the tool is still undergoing further development and has not yet been examined in an efficacy study.

The interviews also highlighted the importance of structured support for deprescribing conversations^13^**.** Enablers for successful deprescribing conversations are information, knowledge and an awareness of indications and risks^14,26,30,31^. While patients rarely initiate the topic of deprescribing, GPs viewed it as their responsibility to bring it up^24,26^—especially given that many patients have a strong attachment to their medications and often lack internal motivation to stop them^23^.

A notable finding was the divergence between the positive perception of the tool’s concept and its limited long-term implementation. While GPs acknowledged its usefulness, its use often declined over time. This suggests that positive evaluations alone do not guarantee sustained integration into clinical practice. Several barriers hindered continued use, including time constraints, a lack of integration into existing systems, technical limitations, and the perceived redundancy after initial familiarisation. This points to a broader challenge in implementing digital decision aids in everyday routines. A systematic review by Rodrigues et al. (2024)^32^ similarly found that usability issues, time demands, and limited integration into clinical workflows commonly hinder long-term adoption. Reflecting on the development of the intervention, it is likely that arriba-PPI could have been more effective if designed in line with current the Medical Research Council (MRC) framework for complex interventions^33^. This would involve preliminary qualitative interviews, literature analysis, and input from an expert panel prior to implementation. Incorporating these steps could enhance both the relevance and usability of complex interventions, and represents an important methodological lesson for future research.

At the same time, the tool’s impact extended beyond its content. The training session provided by the research team were seen as equally—if not more—influential in raising awareness and reducing PPI prescriptions. This highlights the value of direct professional engagement and ongoing education in promoting guideline-based deprescribing practices and in line with the theory of “academic detailing”^34,35^.

In Germany, general practitioners play a central role in long-term medication management, and PPIs are commonly prescribed in primary care. Although healthcare systems differ, challenges related to deprescribing and shared decision-making are widely shared, supporting the transferability of our findings.

For contextualisation, and to contrast professional and patient perspectives, we compared the findings from the GP interviews with results from a separate qualitative study based on patient interviews, which is reported elsewhere^17^. That study explored patients’ experiences with PPI deprescribing and the use of the arriba-PPI tool from the patient perspective. Some patients could no longer remember the tool or stated that it had not been used during the consultation at all^17^. Participation in the study seemed to have a greater influence than the consultation with the tool itself. Patients emphasised the importance of a trusting relationship with their GP and good medical consultation^17^, which is consistent with the results of the GP interviews. They indicated a lack of internal motivation and a need of non-medication alternatives, as previously mentioned. A greater focus on non-pharmacological alternatives could be one strategy to improve the arriba-PPI tool.

### Strengths and limitations

A major strength of this study is the relatively high number of interviews, which enabled the collection of diverse views and experiences. Participants appeared to feel safe and valued, as evidenced by their willingness to share both positive and critical perspectives on the tool. This openness contributed to the richness and credibility of the data.

Among the limitations, the time span between using arriba-PPI and the subsequent interviews varied considerably—from several weeks to nearly a year. This discrepancy may have affected the comparability of responses and introduced recall bias, as participants’ memories and perceptions could have changed over time. However, this variation also provided an opportunity to explore whether the tool had a lasting impact on clinical routines and whether it continued to be used beyond the study period, offering insights into its potential for sustained implementation.

In addition, some interviews were relatively short (e.g. 5–10 min), which limited the opportunity for in-depth probing. This may be attributed to the fact that interviews were conducted by trained but relatively inexperienced research assistants. However, they were closely supervised by experienced researchers throughout the process, which helped ensure consistency and methodological rigor. Even the shorter interviews contributed relevant insights and supported the broad representation of perspectives.

## Conclusion

Structured shared decision-making aids such as arriba-PPI can support prescribing reflection and deprescribing conversations, especially for less experienced clinicians and digitally engaged patients. Yet, long-term use remains limited unless tools are integrated into workflows and combined with non-pharmacological alternatives. For practice, this underlines the need to link digital aids with broader strategies that address patient motivation and holistic care. For research, future efforts should focus on developing comprehensive deprescribing platforms that are co-designed with stakeholders and tested for long-term feasibility. Although this study was conducted in Germany, the findings on enablers and barriers to tool-based deprescribing are likely transferable to other healthcare systems.

## Supplementary Information

Supplementary Information 1.

Supplementary Information 2.

## Data Availability

The data analysed within the study are available from the corresponding author on reasonable request.

## Abbreviations

GERD: Gastroesophageal reflux disease
GP: General practitioner
MRC: Medical research council
NSAID: Non-steroidal anti-inflammatory drug
PPI: Proton pump inhibitor
M1, W1, D1: Pseudonym for GPs from Marburg (M), Witten (W) or Düsseldorf (D)
RCT: Randomised controlled trial
