# Supplementary material for: GP perspectives on a computer-assisted strategy to support PPI deprescribing: a qualitative study
Source: Sci Rep. 2026 Mar 11;16:8730. doi: 10.1038/s41598-026-41970-w (PMC12979569; doi:10.1038/s41598-026-41970-w)
Supplement: Supplementary file 2 — Supplementary Information 2. [file 41598_2026_41970_MOESM2_ESM.docx]

Supplementary Table S2. Individual characteristics of interviewed GPs.

| GP | Interview duration (hh:mm:ss) | arriba-PPI usage (in days)* | Study site | Gender | Age  (years) | Practical experience as GP (years) | Setting | Type of practice | GP practice size | Familiar with arriba | Regular use of arriba |
| --- | --- | --- | --- | --- | --- | --- | --- | --- | --- | --- | --- |
| D1 | 00:07:54 | 201 | Düsseldorf | male | 44 | 10 | urban | group | large | yes | no |
| D2 | 00:11:55 | 235 | Düsseldorf | female | 36 | 3 | rural | group | large | yes | yes |
| D3 | 00:08:23 | 240 | Düsseldorf | ° |  |  |  |  |  |  |  |
| D4 | 00:08:39 | 271 | Düsseldorf | male | 52 | 15 | urban | single | large | no | no |
| D5 | 00:11:25 | 258 | Düsseldorf | ° |  |  |  |  |  |  |  |
| D6 | 00:15:50 | 257 | Düsseldorf | male | 60 | 29 | urban | group | large | no | no |
| D7 | 00:16:40 | 250 | Düsseldorf | female | 62 | 12 | urban | single | medium | yes | yes |
| D8 | 00:10:56 | 216 | Düsseldorf | female | 54 | 19 | n/a | single | large | yes | no |
| M1 | 00:09:48 | 456 | Marburg | male | 58 | 21 | rural | group | large | yes | n/a |
| M2 | 00:05:05 | 489 | Marburg | male | 65 | 32 | rural | single | medium | yes | no |
| M3 | 00:09:11 | 306 | Marburg | male | 60 | 28 | rural | single | small | yes | yes |
| M4 | 00:10:11 | 216 | Marburg | male | 60 | 15 | rural | single | small | no | no |
| M5 | 00:11:31 | 300 | Marburg | male | 56 | 29 | rural | single | medium | yes | yes |
| M6 | 00:09:22 | 460 | Marburg | male | n/a | 20 | rural | group | large | yes | yes |
| M7 | 00:06:33 | 473 | Marburg | male | 54 | 19 | n/a | single | small | yes | no |
| M8 | 00:05:45 | 423 | Marburg | male | 61 | 31 | rural | group | medium | yes | n/a |
| M9 | 00:05:04 | 310 | Marburg | female | 58 | 31 | urban | single | large | yes | no |
| M10 | 00:08:58 | 244 | Marburg | male | 58 | 13 | urban | single | large | yes | no |
| M11 | 00:08:57 | °° | Marburg | male | 54 | 18 | rural | single | medium | yes | no |
| W1 | 00:06:01 | 364 | Witten | female | 57 | 37 | urban | group | medium | no | no |
| W2 | 00:09:17 | 345 | Witten | male | 50 | 18 | urban | group | large | yes | yes |
| W3 | 00:11:02 | 382 | Witten | male | 63 | 33 | urban | group | large | yes | no |
| W4 | 00:18:05 | 389 | Witten | female | 45 | 9 | urban | group | large | yes | no |
| W5 | 00:09:49 | 282 | Witten | male | 61 | 26 | urban | single | medium | yes | yes |
| W6 | 00:17:08 | 296 | Witten | male | 61 | 26 | urban | single | medium | yes | no |
| W7 | 00:07:18 | °° | Witten | male | 47 | 6 | urban | single | medium | no | no |

M: Marburg, W: Witten, D: Düsseldorf; * time (in days) between arriba-PPI training and interview; ° no filled out questionnaire; °° dates missing; n/a: question not answered
